# Performance of the Phoenix Sepsis Score for in-hospital mortality in critically ill pediatric oncology patients with suspected infection

**DOI:** 10.1080/07853890.2026.2743326

**Published:** 2026-10-09

**Authors:** Dianwei Xing, Zijing Xiao, Baiming Liu, Jingjing Xu, Yufeng Liang

**Affiliations:** Guangzhou Women and Children’s Medical Center, Guangzhou Medical University, Guangzhou, Guangdong, China.

**Keywords:** Phoenix Sepsis Score, mortality, pediatric oncology, infection, organ dysfunction

## Abstract

**Background:**

Pediatric oncology patients with infection face a high risk of death. Although the Phoenix Sepsis Score (PSS) has recently been introduced as an organ dysfunction score for pediatric sepsis, its performance in pediatric oncology populations remains insufficiently characterized.

**Methods:**

This retrospective study enrolled pediatric oncology patients admitted to the pediatric intensive care unit (PICU) with suspected infection. The performance of the PSS was evaluated and compared with other pediatric critical care scores for discrimination of in-hospital mortality.

**Results:**

The study cohort comprised 224 patients, accounting for 254 PICU admissions. The overall hospital mortality rate was 17.7%. All scoring systems except the Pediatric Risk of Mortality III (PRISM III) score demonstrated good discrimination. Phoenix-8 showed the highest area under the curve (AUC) (0.855, 95% CI: 0.791–0.919), followed by PSS (0.837) and Pediatric Sequential Organ Failure Assessment (pSOFA) score (0.836). Phoenix-8 had significantly higher AUCs than PHO-Phoenix and PRISM III; differences among the other scoring systems were not statistically significant. Sensitivity analyses were broadly consistent with the primary analysis but showed lower AUCs after inclusion of deaths following discontinuation of life-sustaining therapy.

**Conclusions:**

The PSS showed good discrimination for in-hospital mortality in PICU-admitted pediatric oncology patients with infection, with an AUC of similar magnitude to those of pSOFA and PELOD-2. Its lower data requirements may support its use as a clinically feasible tool for mortality risk assessment in this high-risk population.

## 1. Introduction

Pediatric patients with oncology who are admitted to intensive care for suspected or confirmed infection form a particularly vulnerable and clinically challenging group [1]. Their risk of organ dysfunction reflects both cancer-related factors, such as organ infiltration and immune compromise, and treatment-related factors, including toxicity from systemic therapy, profound and sustained myelosuppression, and invasive procedures such as central venous catheterization [2–5]. Consequently, children undergoing anticancer treatment have a markedly higher risk of death related to sepsis [6,7]. Recognizing those at greatest risk early in the disease course is challenging. The clinical manifestations of infection may overlap with those of cancer progression or chemotherapy-associated organ injury. Several pediatric severity-of-illness and mortality risk scores, including pSOFA, Pediatric Logistic Organ Dysfunction-2 (PELOD-2), and PRISM III score, have been widely used in critically ill children [8–12]. These scores were developed mainly in heterogeneous PICU populations and may not fully capture the distinct pathophysiology of pediatric oncology patients, which may lead to variable or suboptimal predictive performance in this group [6,13,14].

The Society of Critical Care Medicine (SCCM) Pediatric Sepsis Definition Task Force published revised diagnostic standards for pediatric sepsis and septic shock in January 2024 [15]. Drawing on extensive clinical datasets, the task force created the PSS to quantify sepsis-associated organ dysfunction across four key systems: respiratory, cardiovascular, coagulation, and neurological function. In addition, an expanded version covering eight organ systems, the Phoenix-8 score, was introduced [16]. For children with suspected infection, a PSS ≥2 defines sepsis, while sepsis accompanied by a cardiovascular subscore ≥1 is classified as septic shock. Although the PSS has demonstrated good performance in general PICU populations [17–19], its applicability and comparative performance in pediatric oncology patients remain insufficiently investigated. Moreover, most previous studies have focused primarily on discrimination, while evidence integrating calibration and clinical utility in this high-risk subgroup is still limited [13,14].

Accordingly, this study aimed to evaluate the performance of the PSS in critically ill pediatric oncology patients with suspected infection and to compare its performance with established pediatric scoring systems.

## 2. Methods

### 2.1. Study design

This retrospective study included children with oncology who were admitted to the PICU of the Women and Children’s Medical Center, Guangzhou Medical University, between January 2016 and December 2024 for suspected or confirmed infection. For the present study, suspected infection was operationally characterized by the use of systemic antimicrobial therapy and microbiological assessment within 24 h following PICU admission, according to the approach employed during the derivation of the Phoenix Sepsis Score [16]. PICU admissions were excluded when any of the following conditions were present: (1) death occurring within 2 h after PICU admission; (2) death after discontinuation of life-sustaining therapy; (3) discharge against medical advice or at the request of guardians; or (4) missing clinical data exceeding 30%. The unit of analysis was the eligible PICU admission. This exclusion was retained in the primary analysis because deaths following discontinuation of life-sustaining therapy may be influenced by factors beyond acute physiologic severity, including underlying cancer prognosis and goals-of-care decisions. Because exclusion of admissions ending in death after discontinuation of life-sustaining therapy could introduce selection bias, we additionally performed a sensitivity analysis in which these patients were included and classified as in-hospital deaths. Readmissions occurring within 48 h after transfer to a general ward were considered part of the same clinical episode, whereas admissions separated by more than 48 h were considered new admissions. To assess the potential influence of repeated admissions from the same patient, an additional sensitivity analysis was restricted to the chronologically first eligible PICU admission for each patient. All data were extracted from the electronic medical record system and anonymized prior to analysis. Data extraction was performed from November 2025 to January 2026, and statistical analyses were conducted from February 2026 to March 2026. Each eligible admission was identified using a unique admission identifier, and investigators had no access to directly identifiable information during data collection or analysis. A detailed flowchart describing participant inclusion and exclusion is provided in Figure 1.

**Figure 1.**
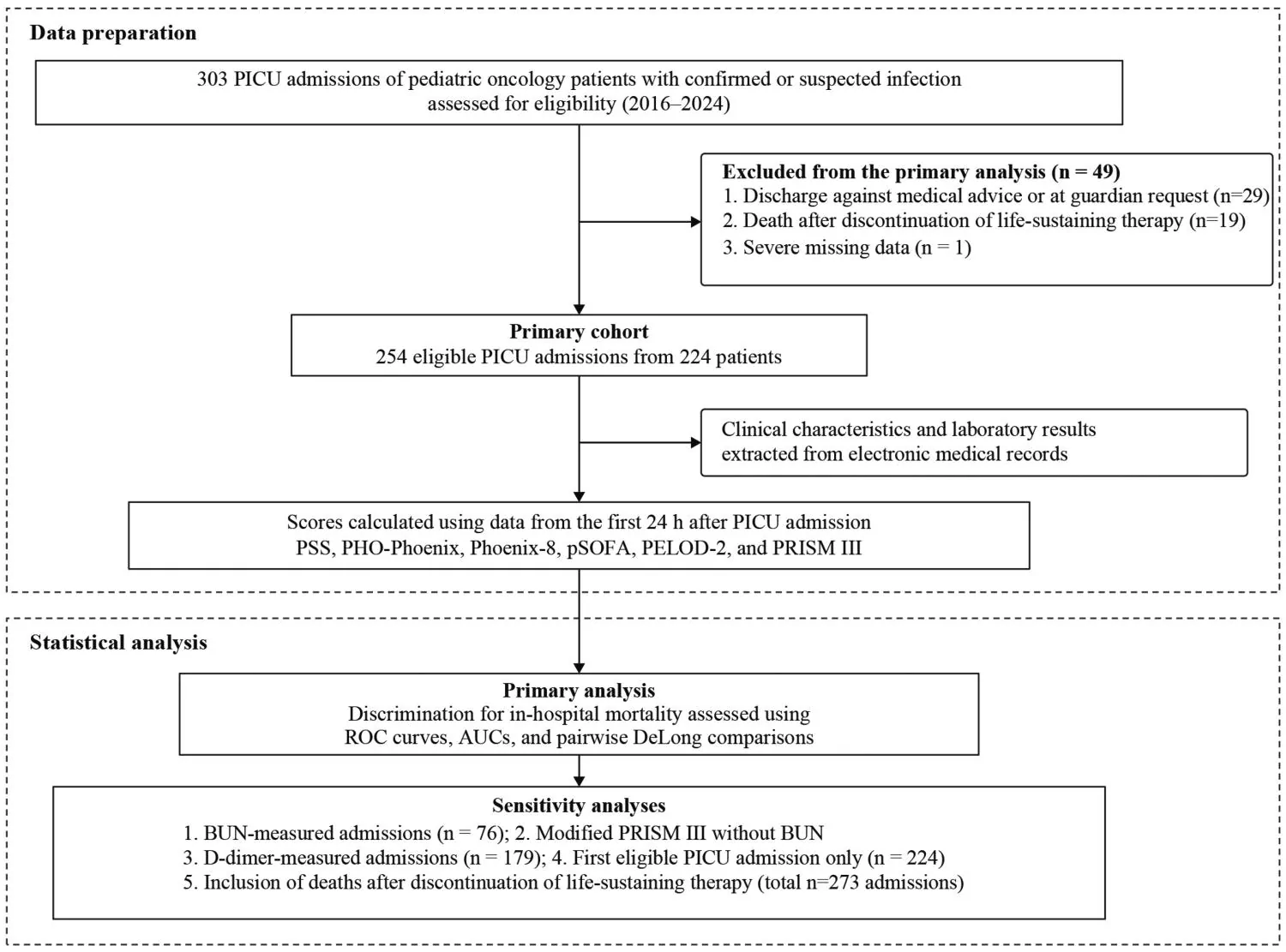
Flowchart of the study.

### 2.2. Data collection

In-hospital mortality served as the primary study endpoint. Data were extracted from the electronic medical record system and included baseline demographic characteristics, clinical information, and outcomes (survival or death), such as sex, age, diagnosis, and admission route. Physiological variables related to organ dysfunction were collected for the calculation of the PSS, Phoenix-8, PHO-Phoenix (without platelet count), pSOFA, PELOD-2, and PRISM III scores. For all scoring systems, the most abnormal values within the first 24 h after PICU admission were used. To enhance data reliability and minimize inconsistencies, data entry was performed by a designated PICU physician and independently supervised by another PICU physician. All data were further reviewed and verified by senior clinicians.

### 2.3. Handling of unmeasured score components

Within the first 24 h after PICU admission, D-dimer was not clinically measured in 75 of 254 admissions (29.5%), and BUN was not clinically measured in 178 of 254 admissions (70.1%). Following the approach used in the development of the PSS, missing variables were treated as normal and assigned 0 points [16]. This strategy is an integral part of the PSS scoring framework. It was designed to avoid artificially increasing a patient’s risk score and to improve the applicability of the score across settings with different resource availability. Accordingly, unmeasured D-dimer values were treated as normal and assigned 0 points in the Phoenix-related scores. For PRISM III, unmeasured BUN likewise did not contribute points in the primary analysis. This approach has been used in previous applications of PRISM III, in which unmeasured physiologic variables were treated as being within the normal range [20]. Because the high proportion of admissions without BUN measurement could influence the estimated performance of PRISM III, two sensitivity analyses were performed. First, all evaluated scores were recalculated and compared within the subset of admissions in which BUN had been measured. Second, a modified PRISM III score was calculated after removing the BUN component for all admissions. A corresponding complete-measurement sensitivity analysis was also performed among admissions with measured D-dimer.

### 2.4. Statistical analysis

This retrospective study included all eligible PICU admissions during the predefined study period. Therefore, no a priori sample-size or power calculation was performed. All statistical analyses were conducted in R software (version 4.4.1). Continuous variables were first tested for normality using the Shapiro–Wilk test. Variables with a normal distribution are expressed as mean ± standard deviation and were compared using independent-samples t-tests. Skewed variables are expressed as median with interquartile range [M (IQR)] and were compared using the Mann–Whitney U test. Categorical variables are summarized as numbers and percentages, with between-group comparisons performed using the chi-square test or Fisher’s exact test. Receiver operating characteristic (ROC) curves were used to evaluate the ability of PSS, pSOFA, PHO-Phoenix, Phoenix-8, PELOD-2, and PRISM III scores to predict in-hospital mortality. The area under the curve (AUC) and corresponding 95% confidence interval (CI) were calculated. Pairwise differences in AUCs were assessed using DeLong’s test. Sensitivity analyses included restriction to admissions with measured BUN or D-dimer, recalculation of PRISM III without the BUN component, inclusion of admissions ending in death after discontinuation of life-sustaining therapy, and restriction to the first eligible PICU admission per patient. Pairwise AUC comparisons were repeated for the two sensitivity analyses that altered cohort composition. For each score, the optimal cutoff was selected according to the maximum Youden index. Sensitivity, specificity, positive predictive value, negative predictive value, and the Youden index were then calculated. For calibration and decision curve analyses, each score was entered separately into a univariable logistic regression model fitted within the study cohort to generate predicted probabilities of in-hospital mortality. Calibration was assessed using calibration plots, the Hosmer–Lemeshow goodness-of-fit test, and the Brier score. Clinical utility was evaluated using decision curve analysis. All statistical tests were two-sided, with statistical significance set at *p* < 0.05.

## 3. Results

### 3.1. Demographic and clinical features of the cohort

A total of 224 unique patients contributed 254 eligible PICU admissions for suspected or confirmed infection. An overview of the cohort’s demographic and clinical data is provided (Table 1). The median age at PICU admission was 56.5 months (IQR, 29.8–109.3 months); 142 (55.9%) were male, and 112 (44.1%) were female. Among the 254 PICU admission episodes, 45 resulted in in-hospital death, corresponding to an in-hospital mortality rate of 17.7%. The median time to death was 2 days (IQR, 1.0–8.5 days). Among the underlying diagnoses, acute lymphoblastic leukemia was the most common (41.3%), followed by myeloid leukemia (24.8%). Lymphoma and solid tumors accounted for 18.5% and 15.4%, respectively. Regarding the admission source, 204 admissions (80.3%) were admitted from the inpatient ward, while 50 admissions (19.7%) were admitted from the emergency department. The distributions of the evaluated severity scores according to in-hospital outcome are provided (Supplementary Figure S1). Of the 254 eligible admissions, 234 (92.1%) had a PSS ≥ 2, 238 (93.7%) had a PELOD-2 ≥ 2, and 247 (97.2%) had a pSOFA ≥ 2.

**Table 1.** Baseline characteristics and clinical scores of the study cohort .

Variables	All (*n* = 254)	Survival (*n* = 209)	Death (*n* = 45)	*p* value
**Characteristics**				
Age (mon)	56.5(29.8, 109.3)	63.0(33.0,111.5)	40.0(25.5,87.5)	0.035
Gender				0.226
Male, n (%)	142(55.9%)	121(57.9%)	21(46.7%)	
Diagnostic group				0.260
Hematologic, n (%)	215(84.6%)			
ALL, n (%)	105(41.3%)	85(40.7%)	20(44.4%)	
Myeloid, n (%)	63(24.8%)	48(23.0%)	15(33.3%)	
Lymphoma, n (%)	47(18.5%)	41(19.6%)	6(13.3%)	
Solid tumor, n (%)	39(15.4%)	35(16.7%)	4(8.9%)	
Organ dysfunction scores, median (IQR)				
PSS	4(3,6)	3(3,5)	7(5,9)	<0.001
PHO-Phoenix	3(2,5)	3(2,5)	6(5,9)	<0.001
Phoenix-8	5(4,7)	5(3,6)	9(7,11)	<0.001
pSOFA	8(5,10)	7(4,9)	12(10,13)	<0.001
PELOD-2	6(4,9)	6(4,8)	10(8,14)	<0.001
PRISM III	11(7,14)	10(6,13)	15(12,21)	<0.001
Admission location				0.330
Emergency department, n (%)	50(19.7%)	44 (21.1%)	6(13.3%)	
Inpatient, n (%)	204(80.3%)	165 (78.9%)	39(86.7%)	

### 3.2. Discriminative performance of the scoring systems

The discriminatory performance of the evaluated scoring systems was assessed using ROC analysis (Figure 2), and the resulting AUC estimates with 95% confidence intervals are summarized (Figure 3). Except for PRISM III, all scoring systems demonstrated good discriminatory performance for predicting in-hospital mortality (AUC > 0.80). Phoenix-8 achieved the highest AUC of 0.855 (95% CI: 0.791–0.919), followed by PSS (0.837, 95% CI: 0.770–0.904) and pSOFA (0.836, 95% CI: 0.765–0.908). The AUCs of PHO-Phoenix and PELOD-2 were 0.824 (95% CI: 0.756–0.892) and 0.817 (95% CI: 0.746–0.889), respectively. PRISM III showed the lowest AUC of 0.783 (95% CI: 0.707–0.859). DeLong’s test was used for pairwise comparisons of AUCs (Supplementary Table S1). Phoenix-8 had a significantly higher AUC than PHO-Phoenix (ΔAUC = 0.031, 95% CI: 0.002–0.060; *p* = 0.038) and PRISM III (ΔAUC = 0.072, 95% CI: 0.010–0.133; *p* = 0.022). No statistically significant differences were observed in the remaining pairwise comparisons (all *p* > 0.05).

**Figure 2.**
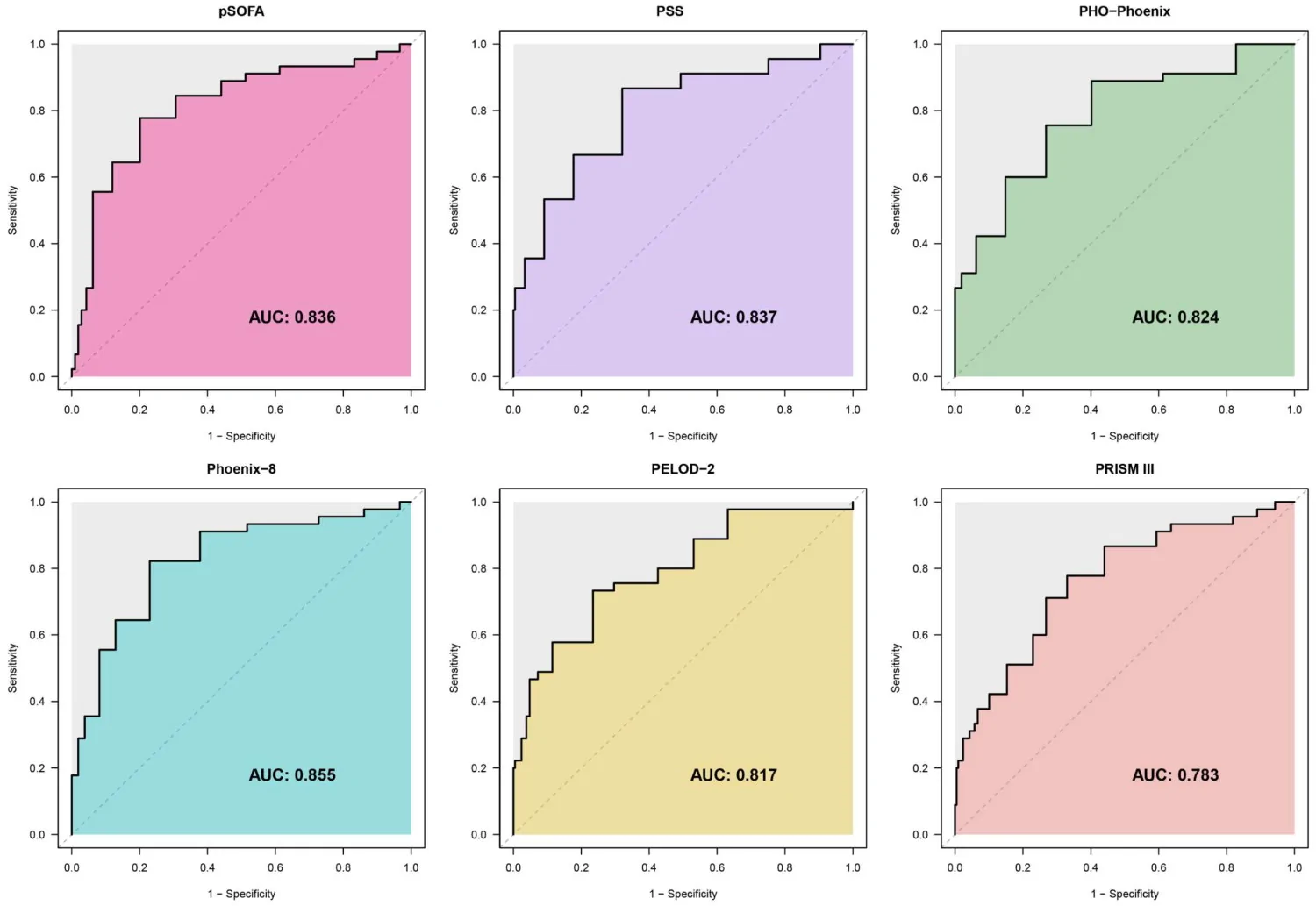
ROC curves of pSOFA, PSS, PHO-Phoenix, Phoenix-8, PELOD-2, and PRISM III for predicting in-hospital mortality in pediatric oncology patients admitted to the PICU with suspected infection.

**Figure 3.**
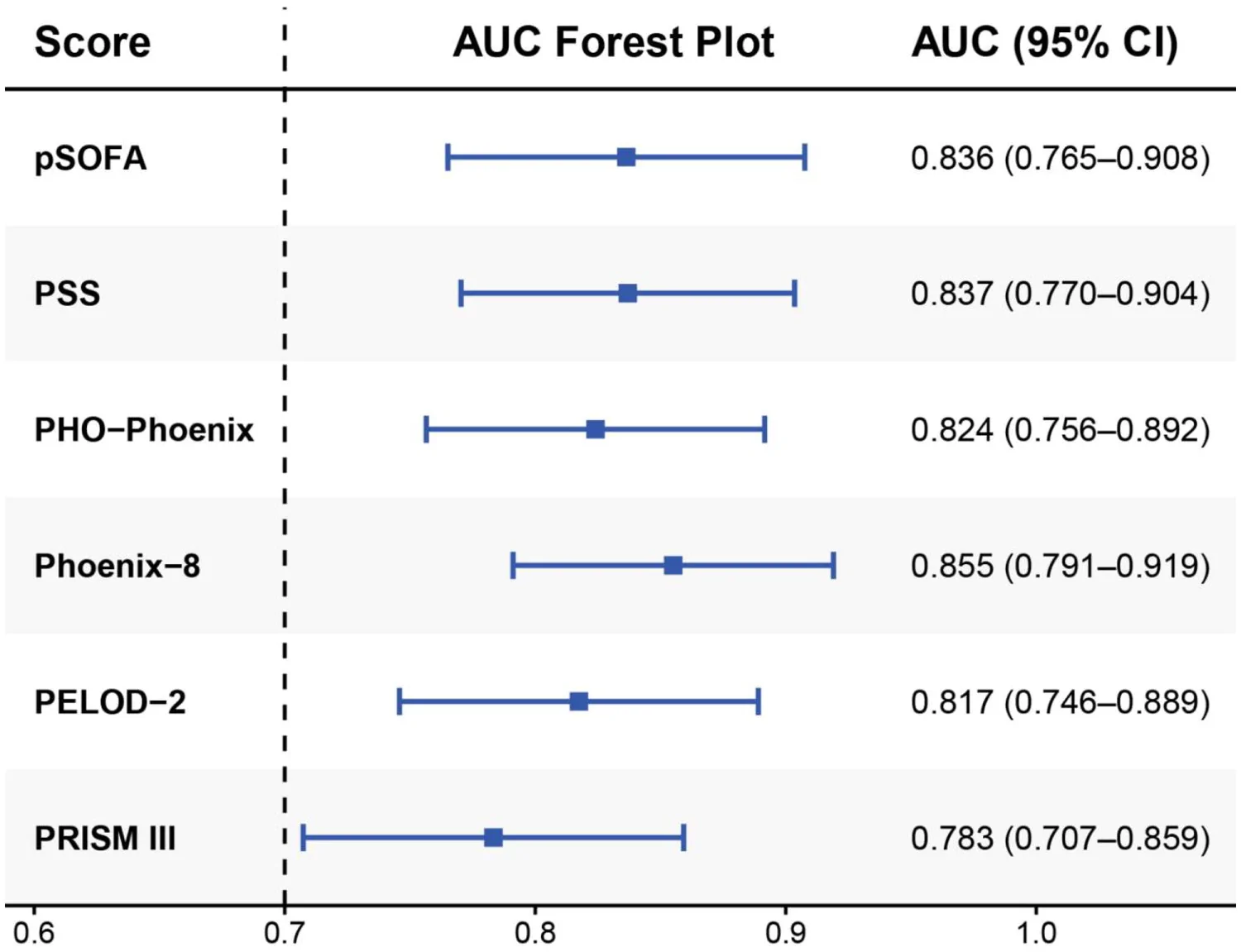
Forest plot of the AUC and corresponding 95% CI for the evaluated scoring systems.

### 3.3. Optimal cutoff values and classification performance

Using the maximum Youden index, the optimal thresholds were identified as ≥10 for pSOFA, ≥5 for PSS, ≥5 for PHO-Phoenix, ≥7 for Phoenix-8, ≥9 for PELOD-2, and ≥12 for PRISM III (Table 2). Among these, Phoenix-8 showed the highest Youden index (0.593) among the evaluated scores at the cohort-derived optimal thresholds. PSS demonstrated the highest sensitivity (86.7%) and negative predictive value (95.9%). All scoring systems demonstrated high negative predictive values (all ≥ 93%).

**Table 2.** Optimal cut-off values and diagnostic performance of pediatric severity scores for predicting in-hospital mortality.

Score	Clinical cut-off	Sensitivity	Specificity	PPV	NPV	Youden Index
pSOFA	≥10	77.8%	79.9%	45.5%	94.4%	0.577
PSS	≥5	86.7%	67.9%	36.8%	95.9%	0.546
PHO-Phoenix	≥5	75.6%	73.2%	37.8%	93.3%	0.488
Phoenix-8	≥7	82.2%	77.0%	43.5%	95.3%	0.593
PELOD-2	≥9	73.3%	76.6%	40.2%	93.0%	0.499
PRISM III	≥12	77.8%	67.0%	33.7%	93.3%	0.448

### 3.4. Calibration analysis of the scoring systems

Calibration within the study cohort was evaluated using calibration plots, Hosmer–Lemeshow goodness-of-fit tests, and Brier scores (Figure 4, Supplementary Table S2). No significant lack of fit was detected by the Hosmer–Lemeshow test across the evaluated scoring systems (all *p* > 0.05). Brier scores ranged from 0.100 to 0.116, with Phoenix-8 showing the lowest value (0.100), followed by PSS (0.102), PHO-Phoenix (0.104), PELOD-2 (0.105), pSOFA (0.108), and PRISM III (0.116). Calibration curves were broadly consistent with the Brier score results. Calibration plots showed generally acceptable within-cohort agreement between predicted and observed mortality, although PRISM III demonstrated some deviation in the higher-risk range.

**Figure 4.**
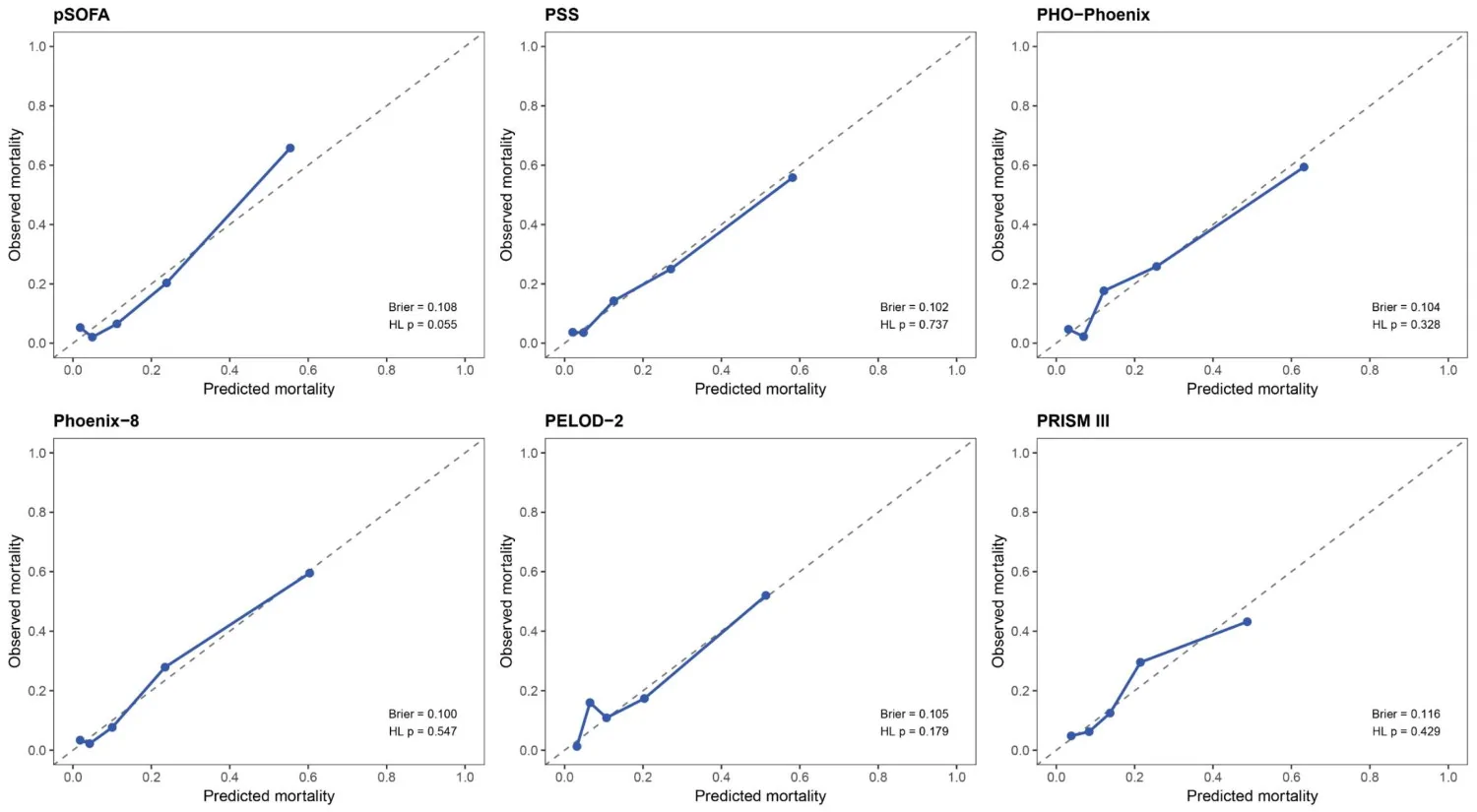
Calibration plots for pSOFA, PSS, PHO–Phoenix, Phoenix–8, PELOD-2, and PRISM III in predicting in-hospital mortality among pediatric oncology patients admitted to the PICU with suspected infection. Brier scores and Hosmer–Lemeshow (HL) goodness-of-fit test results are shown for each scoring system.

### 3.5. Decision curve analysis for assessing clinical applicability

Results from the decision curve analysis showed that the evaluated scores maintained positive clinical utility throughout a clinically meaningful range of threshold probabilities (Figure 5). Phoenix-8 demonstrated marginally greater net benefit at lower threshold probabilities (10%–20%), whereas pSOFA demonstrated the highest net benefit within the 20%–40% range. PSS, PHO-Phoenix, and PELOD-2 showed stable performance across the assessed threshold range, whereas PRISM III provided the lowest net benefit over most thresholds.

**Figure 5.**
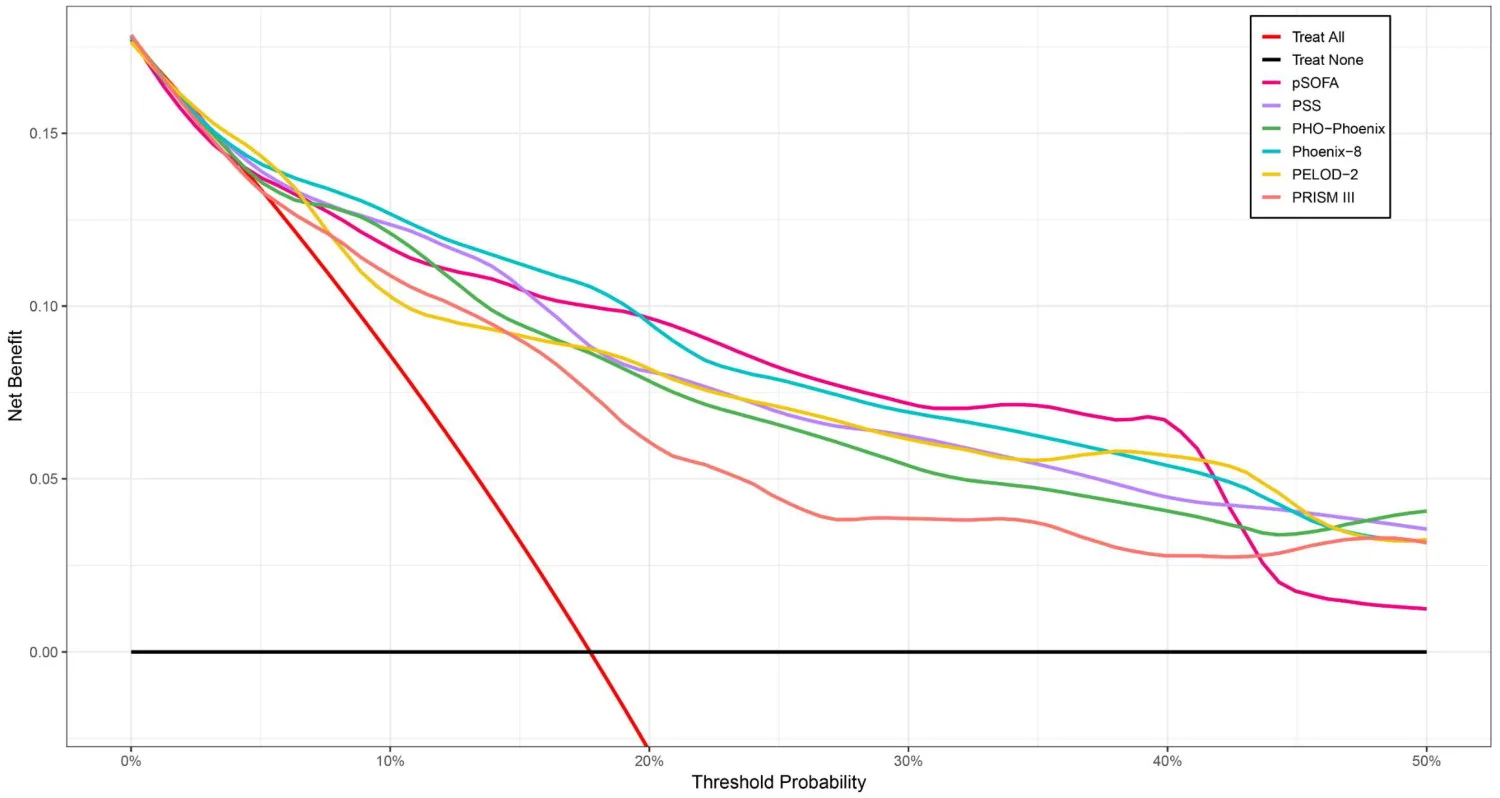
Decision curve analysis of the evaluated scoring systems for predicting in-hospital mortality.

### 3.6. Sensitivity analyses

Sensitivity analyses were broadly consistent with the primary analysis, although some pairwise comparisons differed (Supplementary Tables S3–S5). Among admissions with measured BUN (*n* = 76; 17 deaths), the AUC was 0.801 (95% CI, 0.684–0.917) for PRISM III and 0.855 (95% CI, 0.768–0.942) for Phoenix-8. The difference between the two scores was no longer statistically significant (ΔAUC = 0.054; 95% CI, −0.044 to 0.153; *p* = 0.281). After removal of the BUN component, the AUC of PRISM III was 0.770 (95% CI, 0.691–0.848). Among admissions with measured D-dimer (*n* = 179; 32 deaths), discrimination was similar to that in the primary analysis, with an AUC of 0.853 (95% CI, 0.780–0.927) for Phoenix-8. When the 19 additional admissions ending in death after discontinuation of life-sustaining therapy were included, the AUCs of all scores were lower than in the primary analysis. Phoenix-8 retained the highest AUC point estimate (0.803; 95% CI, 0.740–0.865). When the analysis was restricted to the first eligible PICU admission per patient, overall discrimination remained similar to the primary analysis, although some pairwise comparisons changed. The ΔAUC between Phoenix-8 and PRISM III decreased from 0.072 in the primary analysis to 0.045 and was no longer statistically significant (95% CI, −0.014 to 0.105; *p* = 0.136).

## 4. Discussion

In this study, the PSS showed good discrimination for in-hospital mortality among critically ill pediatric oncology patients with suspected infection, with performance similar in magnitude to pSOFA and PELOD-2. Phoenix-8 yielded the highest AUC point estimate in the primary analysis, although its differences from PSS, pSOFA, and PELOD-2 were not statistically significant. Sensitivity analyses broadly supported the overall pattern of these findings. Removing platelet count from the PSS did not improve its performance, whereas PRISM III showed relatively weaker overall performance.

The PSS provides a standardized framework for identifying infection-associated organ dysfunction in pediatric sepsis, with cardiovascular dysfunction playing a particularly important role in the recognition of septic shock [15]. This emphasis is supported by recent mechanistic evidence showing that sepsis-induced vasoplegia is closely related to vascular smooth muscle cell dysfunction, including phenotypic switching and impaired calcium homeostasis, which contribute to loss of vascular tone and circulatory failure [21]. Our findings showed that PSS performed well in identifying patients at risk of death in this pediatric oncology cohort, particularly because the optimal threshold provided high sensitivity and negative predictive value. This supports its potential role in both mortality risk prediction and early clinical risk stratification. Still, its discriminatory performance was almost identical to that of pSOFA in DeLong’s test, and PSS did not demonstrate a statistically significant advantage over established organ dysfunction scores in this cohort. Given its smaller variable set and lower reliance on complex laboratory tests, PSS may be more feasible in resource-limited settings or when data availability is constrained.

The present findings align with some previous reports but differ from others. An American PICU-based investigation of children with oncology showed that the ability of various sepsis and organ dysfunction scoring models to identify mortality risk was limited [13]. Similarly, Wösten-van Asperen et al. reported limited discrimination for 90-day all-cause mortality in a European multicenter cohort of pediatric oncology patients, with AUROCs of 0.66 for PSS, 0.65 for Phoenix-8, 0.64 for PELOD-2, and 0.67 for pSOFA. Discrimination was somewhat higher for PICU mortality, with AUROCs of approximately 0.70–0.72 [14]. By contrast, studies from China and Israel suggested that pSOFA and PELOD-2 had good predictive performance for mortality in children with hematological malignancies or infection after hematopoietic stem cell transplantation [22,23]. One possible explanation for these discrepant findings is the temporal relationship between score assessment and the mortality endpoint. In the European cohort, the median time to death was 12.5 days, whereas it was 2 days in our cohort. Because these scores are calculated from organ dysfunction within the first 24 h after PICU admission, their discrimination may be greater for deaths occurring closer to the time of assessment, while later mortality may be increasingly influenced by secondary infections, PICU-related complications, progression of the underlying malignancy, and treatment-related toxicity. Differences across studies in inclusion criteria, tumor composition, infection definitions, timing of PICU admission, mortality endpoints, and treatment resources may also have influenced the external performance of these scoring systems.

In this study, Phoenix-8 achieved the highest discrimination, and its performance across the evaluation metrics followed the same overall pattern. Because Phoenix-8 incorporates more organ systems than PSS, it may provide a more complete assessment of multisystem involvement in infection-related critical illness. This broader assessment may be better suited to pediatric oncology patients, in whom chemotherapy-related immunosuppression and myelosuppression increase susceptibility to infection and may accelerate progression to multiple organ dysfunction [5]. However, the pairwise comparisons should be interpreted cautiously. Although Phoenix-8 had significantly higher AUCs than PHO-Phoenix and PRISM III in the primary cohort, the statistical significance of individual comparisons changed in some sensitivity analyses. Thus, the present findings support favorable discrimination of Phoenix-8 but do not establish consistent statistical superiority over all comparator scores.

Unlike Phoenix-8, PHO-Phoenix did not outperform PSS after the removal of platelet count, a finding consistent with Wolf J. et al. [13]. In children undergoing cancer treatment, low platelet counts may be attributable to chemotherapy exposure, bone marrow disease, infectious complications, transfusion-related factors, or the underlying neoplasm. The relationship between platelet count and infection-related mortality may therefore be influenced by several non-infectious factors. These findings suggest that optimizing scoring systems for pediatric oncology patients should not rely solely on a single hematological variable, but should place greater emphasis on multisystem organ dysfunction and dynamic changes in clinical status.

Sensitivity analyses further supported the overall pattern of the primary findings, while also showing that some results were influenced by data completeness and cohort composition. Among admissions with measured D-dimer, discrimination was broadly similar to that in the primary analysis, suggesting that the absence of D-dimer measurements had limited influence on the overall findings. This is consistent with the development and validation studies of the Phoenix scores, which showed good discrimination even in centers where D-dimer and fibrinogen were not routinely measured [15,16]. PRISM III showed only a modest increase in discrimination among admissions with measured BUN, and its performance did not improve after the BUN component was removed. Given the approach used in previous applications of PRISM III to handle unmeasured physiologic variables [20], the lower AUC observed in the primary analysis is unlikely to be explained solely by the absence of BUN measurements. Including admissions ending in death after discontinuation of life-sustaining therapy reduced the AUCs of all scores, suggesting that their exclusion from the primary analysis may have led to somewhat higher estimates of absolute discrimination. Restricting the analysis to the first eligible PICU admission per patient produced broadly similar AUCs, although the magnitude and statistical significance of some pairwise differences changed. The relative performance of the scoring systems should therefore be interpreted cautiously.

The findings of this study may have practical clinical value. First, they provide additional performance data for the PSS in PICU-admitted pediatric oncology patients with infection. Second, PSS showed discrimination of a similar magnitude to pSOFA and PELOD-2 while requiring fewer data elements, making it potentially more feasible in settings with limited resources. Third, Phoenix-8 showed favorable discrimination and also performed well in the calibration and decision curve analyses, suggesting that broader assessment of organ dysfunction may provide useful information for mortality risk stratification in this population. Finally, the high negative predictive values observed at the cohort-derived cutoffs suggest potential value for identifying patients at lower short-term mortality risk.

The present study is subject to several constraints. First, its retrospective design and single-center setting, together with the prolonged enrollment period, may limit the generalizability of the findings. Changes in PICU care, antimicrobial treatment, oncological therapy, and supportive management over time may have influenced the results. Second, the overall sample size and number of in-hospital deaths were limited, which reduced the precision of some pairwise AUC comparisons. Changes in nominal statistical significance across sensitivity analyses should therefore be interpreted cautiously and should not be considered evidence of equivalence or definitive superiority between scores. Third, some score components were not clinically measured within the scoring window, particularly BUN for PRISM III and D-dimer for the Phoenix-related scores. Although targeted sensitivity analyses were performed, the BUN-measured subset represented a clinically selected subgroup and may have differed in case mix from the overall cohort. Fourth, admissions ending in death after discontinuation of life-sustaining therapy were excluded from the primary cohort. Their inclusion in a sensitivity analysis reduced the AUCs of all evaluated scores, indicating that the primary analysis may have yielded somewhat higher estimates of absolute discrimination. Fifth, some patients contributed more than one eligible PICU admission. Although restriction to the first eligible admission yielded broadly similar discrimination estimates, repeated observations were retained in the primary analysis. Sixth, all scores were calculated using data from the first 24 h after PICU admission, and dynamic changes were not assessed. Complete 90-day follow-up was unavailable, precluding evaluation of score performance for 90-day mortality. Finally, the study was conducted at a single center, and the observed performance estimates have not been confirmed in independent multicenter cohorts.

## 5. Conclusion

In conclusion, the PSS demonstrated good discrimination for in-hospital mortality among PICU-admitted pediatric oncology patients with suspected infection. Its AUC was similar in magnitude to those of pSOFA and PELOD-2. Phoenix-8 consistently showed a high AUC point estimate, although evidence of statistical superiority over individual comparator scores was not uniform across sensitivity analyses. Further prospective, multicenter studies should incorporate dynamic scoring, treatment exposure, and tumor characteristics to improve risk assessment in critically ill children with cancer and infection.

## Supplementary Material

Supplementary Material_revision clean.docx

## Data Availability

The data utilized in this study are available from the corresponding author upon request.

## References

[1] Zinter MS, DuBois SG, Spicer A, et al. Pediatric cancer type predicts infection rate, need for critical care intervention, and mortality in the pediatric intensive care unit. Intensive Care Med. 2014;40(10):1536–1544. doi: 10.1007/s00134-014-3389-2. PMC4177269 25023526

[2] Heying R, Schneider DT, Körholz D, et al. Efficacy and outcome of intensive care in pediatric oncologic patients. Crit Care Med. 2001;29(12):2276–2280. doi: 10.1097/00003246-200112000-00007. 11801824

[3] Heney D, Lewis IJ, Lockwood L, et al. The intensive care unit in paediatric oncology. Arch Dis Child. 1992;67(3):294–298. doi: 10.1136/adc.67.3.294. PMC1793666 1575551

[4] Tamburro R. Pediatric cancer patients in clinical trials of sepsis: factors that predispose to sepsis and stratify outcome. Pediatr Crit Care Med. 2005;6(3 Suppl):S87–S91. doi: 10.1097/01.PCC.0000161288.00396.49. 15857565

[5] Loeffen EAH, Knops RRG, Boerhof J, et al. Treatment-related mortality in children with cancer: prevalence and risk factors. Eur J Cancer. 2019;121:113–122. doi: 10.1016/j.ejca.2019.08.008. 31569066

[6] Rubnitz Z, Sun Y, Agulnik A, et al. Prediction of attributable mortality in pediatric patients with cancer admitted to the intensive care unit for suspected infection: a comprehensive evaluation of risk scores. Cancer Med. 2023;12(23):21287–21292. doi: 10.1002/cam4.6709. PMC10726759 38011018

[7] Aljabari S, Balch A, Larsen GY, et al. Severe sepsis-associated morbidity and mortality among critically ill children with cancer. J Pediatr Intensive Care. 2019;8(3):122–129. doi: 10.1055/s-0038-1676658. PMC6687451 31404226

[8] Menon K, Schlapbach LJ, Akech S, et al. Criteria for pediatric sepsis – a systematic review and meta-analysis by the Pediatric Sepsis Definition Taskforce. Crit Care Med. 2022;50(1):21–36. doi: 10.1097/CCM.0000000000005294. PMC8670345 34612847

[9] Leteurtre S, Duhamel A, Salleron J, et al. PELOD-2: an update of the PEdiatric logistic organ dysfunction score. Crit Care Med. 2013;41(7):1761–1773. doi: 10.1097/CCM.0b013e31828a2bbd. 23685639

[10] Matics TJ, Sanchez-Pinto LN. Adaptation and validation of a pediatric sequential organ failure assessment score and evaluation of the Sepsis-3 definitions in critically ill children. JAMA Pediatr. 2017;171(10):e172352. doi: 10.1001/jamapediatrics.2017.2352. PMC6583375 28783810

[11] Gonçalves J-P, Severo M, Rocha C, et al. Performance of PRISM III and PELOD-2 scores in a pediatric intensive care unit. Eur J Pediatr. 2015;174(10):1305–1310. doi: 10.1007/s00431-015-2533-5. 25875250

[12] Schlapbach LJ, Straney L, Bellomo R, et al. Prognostic accuracy of age-adapted SOFA, SIRS, PELOD-2, and qSOFA for in-hospital mortality among children with suspected infection admitted to the intensive care unit. Intensive Care Med. 2018;44(2):179–188. doi: 10.1007/s00134-017-5021-8. PMC5816088 29256116

[13] Wolf J, Rubnitz Z, Agulnik A, et al. Phoenix sepsis score and risk of attributable mortality in children with cancer. JAMA Netw Open. 2024;7(6):e2415917. doi: 10.1001/jamanetworkopen.2024.15917. PMC11165374 38857052

[14] Wösten-van Asperen RM, la Roi-Teeuw HM, Tissing WJE, et al. The Phoenix Sepsis Score in pediatric oncology patients with sepsis at PICU admission: test of performance in a European Multicenter Cohort, 2018–2020. Pediatr Crit Care Med. 2025;26(2):e177–e185. doi: 10.1097/PCC.0000000000003683. PMC11792984 39982155

[15] Schlapbach LJ, Watson RS, Sorce LR, et al. International consensus criteria for pediatric sepsis and septic shock. JAMA. 2024;331(8):665–674. doi: 10.1001/jama.2024.0179. PMC10900966 38245889

[16] Sanchez-Pinto LN, Bennett TD, DeWitt PE, et al. Development and validation of the phoenix criteria for pediatric sepsis and septic shock. JAMA. 2024;331(8):675–686. doi: 10.1001/jama.2024.0196. PMC10900964 38245897

[17] Sanchez-Pinto LN, Daniels LA, Atreya M, et al. Phoenix sepsis criteria in critically ill children: retrospective validation using a United States Nine-Center Dataset, 2012–2018. Pediatr Crit Care Med. 2025;26(2):e155–e165. doi: 10.1097/PCC.0000000000003675. PMC11792981 39982153

[18] Xing D, Xiao Z, Liu B, et al. Comparison of Phoenix Sepsis Score and traditional scoring systems in predicting mortality in PICU patients with suspected infection. Sci Rep. 2026;16(1):16060. doi: 10.1038/s41598-026-47381-1. PMC13199530 41933032

[19] Marchetto L, Daverio M, Comoretto R, et al. Predictive and Prognostic Performance of the Phoenix Sepsis Criteria and Phoenix Sepsis Score in PICU patients with suspected infection: a multicenter prospective study. Crit Care Med. 2026;54(6):1329–1340. doi: 10.1097/CCM.0000000000007034. PMC13185717 41532812

[20] Ghanad Poor N, West NC, Sreepada RS, et al. An Artificial Neural Network-Based Pediatric Mortality Risk Score: development and performance evaluation using data from a Large North American Registry. JMIR Med Inform. 2021;9(8):e24079. doi: 10.2196/24079. PMC8441599 34463636

[21] Ruan H, Shen X-Y, Liu S-Y, et al. Role of vascular smooth muscle cell pathobiology in sepsis‑induced vasoplegia (Review). Int J Mol Med. 2026;57(4):87. doi: 10.3892/ijmm.2026.5758. PMC12900270 41645740

[22] Wittmann Dayagi T, Nirel R, Avrahami G, et al. A need for a novel survival risk scoring system for intensive care admissions due to sepsis in pediatric hematology/oncology patients. J Intensive Care Med. 2024;39(5):484–492. doi: 10.1177/08850666231216362. 37981801

[23] Wu L, Jin M, Wang R, et al. Prognostic factors of sepsis in children with acute leukemia admitted to the pediatric intensive care unit. Pediatr Blood Cancer. 2023;70:e30382. doi: 10.1002/pbc.30382. 37283279

