# Supplementary material for: Performance of the Phoenix Sepsis Score for in-hospital mortality in critically ill pediatric oncology patients with suspected infection
Source: Ann Med. 2026 Oct 9;58(1):2743326. doi: 10.1080/07853890.2026.2743326 (PMC13656676; doi:10.1080/07853890.2026.2743326)
Supplement: Supplementary file 1 [file IANN_A_2743326_SM0384.docx]

Supplementary Material

# Supplementary Figure

**Supplementary Figure S1** Mortality distribution by PSS, PHO-Phoenix, Phoenix-8, PELOD-2, pSOFA, and PRISM III scores within the first 24 hours after PICU admission among 254 eligible PICU admissions for suspected infection. Numbers above the bars represent the number of admissions at each score value.

**Supplementary Table S1** Pairwise comparisons of prognostic scores for in-hospital mortality using DeLong’s test.

| **Comparison** | **ΔAUC** | **95% CI** | **p value** |
| --- | --- | --- | --- |
| pSOFA vs PSS | -0.001 | -0.047 to 0.046 | 0.980 |
| pSOFA vs PHO-Phoenix | 0.012 | -0.038 to 0.063 | 0.634 |
| pSOFA vs Phoenix-8 | -0.019 | -0.063 to 0.025 | 0.403 |
| pSOFA vs PELOD-2 | 0.019 | -0.037 to 0.075 | 0.510 |
| pSOFA vs PRISM III | 0.053 | -0.010 to 0.116 | 0.098 |
| PSS vs PHO-Phoenix | 0.013 | -0.004 to 0.030 | 0.134 |
| PSS vs Phoenix-8 | -0.018 | -0.040 to 0.004 | 0.109 |
| PSS vs PELOD-2 | 0.020 | -0.024 to 0.063 | 0.383 |
| PSS vs PRISM III | 0.054 | -0.009 to 0.116 | 0.092 |
| PHO-Phoenix vs Phoenix-8 | -0.031 | -0.060 to -0.002 | 0.038 |
| PHO-Phoenix vs PELOD-2 | 0.007 | -0.043 to 0.057 | 0.796 |
| PHO-Phoenix vs PRISM III | 0.041 | -0.027 to 0.109 | 0.241 |
| Phoenix-8 vs PELOD-2 | 0.038 | -0.011 to 0.087 | 0.133 |
| Phoenix-8 vs PRISM III | 0.072 | 0.010 to 0.133 | 0.022 |
| PELOD-2 vs PRISM III | 0.034 | -0.020 to 0.088 | 0.213 |

**Supplementary Table S2** Calibration performance of six prognostic scores for predicting in-hospital mortality.

| **Score** | **Brier Score** | **HL χ²** | **HL p value** |
| --- | --- | --- | --- |
| pSOFA | 0.108 | 7.597 | 0.055 |
| PSS | 0.102 | 1.266 | 0.737 |
| PHO-Phoenix | 0.104 | 3.443 | 0.328 |
| Phoenix-8 | 0.100 | 2.124 | 0.547 |
| PELOD-2 | 0.105 | 4.899 | 0.179 |
| PRISM III | 0.116 | 2.765 | 0.429 |

**Supplementary Table S3**. Sensitivity analyses addressing unmeasured BUN and D-dimer

Panel A. Discriminatory performance in the primary and measurement-restricted cohorts

| **Score** | **Primary cohort n = 254; deaths = 45** | **BUN measured n = 76; deaths = 17** | **D-dimer measured n = 179; deaths = 32** |
| --- | --- | --- | --- |
| pSOFA | 0.836 (0.765 to 0.908) | 0.909 (0.844 to 0.973) | 0.827 (0.740 to 0.913) |
| PSS | 0.837 (0.770 to 0.904) | 0.821 (0.710 to 0.932) | 0.822 (0.738 to 0.907) |
| PHO-Phoenix | 0.824 (0.756 to 0.892) | 0.794 (0.673 to 0.914) | 0.805 (0.715 to 0.895) |
| Phoenix-8 | 0.855 (0.791 to 0.919) | 0.855 (0.768 to 0.942) | 0.853 (0.780 to 0.927) |
| PELOD-2 | 0.817 (0.746 to 0.889) | 0.855 (0.747 to 0.963) | 0.814 (0.724 to 0.905) |
| PRISM III | 0.783 (0.707 to 0.859) | 0.801 (0.684 to 0.917) | 0.792 (0.700 to 0.884) |

Panel B. Phoenix-8 versus PRISM III

| **Analysis** | **ΔAUC (95% CI)** | **p value** |
| --- | --- | --- |
| Primary cohort | 0.072 (0.010 to 0.133) | 0.022 |
| BUN measured | 0.054 (−0.044 to 0.153) | 0.281 |
| D-dimer measured | 0.062 (−0.015 to 0.138) | 0.114 |

Panel C. Modified PRISM III without BUN

| **PRISM III version** | **PRISM III AUC (95% CI)** | **Phoenix-8 AUC (95% CI)** | **ΔAUC (95% CI)** | **p value** |
| --- | --- | --- | --- | --- |
| Original | 0.783 (0.707 to 0.859) | 0.855 (0.791 to 0.919) | 0.072 (0.010 to 0.133) | 0.022 |
| Modified without BUN | 0.770 (0.691 to 0.848) | 0.855 (0.791 to 0.919) | 0.085 (0.023 to 0.147) | 0.007 |

Values in Panel A are AUCs (95% CIs). BUN and D-dimer restrictions were applied separately. Both Panel C analyses used the primary cohort (254 admissions; 45 deaths); modified PRISM III was calculated by subtracting the BUN contribution, with the other scores unchanged. In Panels B and C, ΔAUC is Phoenix-8 minus the indicated PRISM III version.

**Supplementary Table S4** Discriminatory performance according to cohort definition

| **Score** | **Primary cohort n = 254; deaths = 45** | **Expanded cohort**  **n = 273; deaths = 64** | **First eligible admission only n = 224; deaths = 40** |
| --- | --- | --- | --- |
| pSOFA | 0.836 (0.765 to 0.908) | 0.774 (0.704 to 0.845) | 0.859 (0.793 to 0.925) |
| PSS | 0.837 (0.770 to 0.904) | 0.770 (0.702 to 0.837) | 0.841 (0.773 to 0.908) |
| PHO-Phoenix | 0.824 (0.756 to 0.892) | 0.752 (0.681 to 0.822) | 0.821 (0.749 to 0.893) |
| Phoenix-8 | 0.855 (0.791 to 0.919) | 0.803 (0.740 to 0.865) | 0.860 (0.796 to 0.924) |
| PELOD-2 | 0.817 (0.746 to 0.889) | 0.777 (0.711 to 0.842) | 0.841 (0.776 to 0.906) |
| PRISM III | 0.783 (0.707 to 0.859) | 0.733 (0.660 to 0.805) | 0.814 (0.746 to 0.882) |

Values are AUCs (95% CIs). The expanded cohort included the primary 254 admissions and 19 additional admissions with death after discontinuation of life-sustaining therapy; all 19 were classified as deaths. The first-admission-only analysis retained each patient’s chronologically first eligible admission within the primary cohort.

**Supplementary Table S5** Complete pairwise DeLong comparisons according to cohort definition

Panel A. Expanded cohort (n = 273 admissions; 64 deaths)

| **Comparison** | **ΔAUC** | **95% CI** | **p value** |
| --- | --- | --- | --- |
| pSOFA vs PSS | 0.004 | −0.044 to 0.053 | 0.862 |
| pSOFA vs PHO-Phoenix | 0.022 | −0.032 to 0.077 | 0.423 |
| pSOFA vs Phoenix-8 | −0.029 | −0.073 to 0.016 | 0.208 |
| pSOFA vs PELOD-2 | −0.002 | −0.057 to 0.052 | 0.930 |
| pSOFA vs PRISM III | 0.041 | −0.018 to 0.101 | 0.173 |
| PSS vs PHO-Phoenix | 0.018 | 0.002 to 0.035 | 0.032 |
| PSS vs Phoenix-8 | −0.033 | −0.057 to −0.009 | 0.007 |
| PSS vs PELOD-2 | −0.007 | −0.055 to 0.041 | 0.784 |
| PSS vs PRISM III | 0.037 | −0.029 to 0.103 | 0.271 |
| PHO-Phoenix vs Phoenix-8 | −0.051 | −0.082 to −0.020 | 0.001 |
| PHO-Phoenix vs PELOD-2 | −0.025 | −0.079 to 0.029 | 0.370 |
| PHO-Phoenix vs PRISM III | 0.019 | −0.054 to 0.092 | 0.610 |
| Phoenix-8 vs PELOD-2 | 0.026 | −0.022 to 0.074 | 0.283 |
| Phoenix-8 vs PRISM III | 0.070 | 0.009 to 0.131 | 0.025 |
| PELOD-2 vs PRISM III | 0.044 | −0.009 to 0.096 | 0.103 |

Panel B. First eligible admission only (n = 224 admissions; 40 deaths)

| **Comparison** | **ΔAUC** | **95% CI** | **p value** |
| --- | --- | --- | --- |
| pSOFA vs PSS | 0.018 | −0.031 to 0.068 | 0.462 |
| pSOFA vs PHO-Phoenix | 0.038 | −0.015 to 0.091 | 0.163 |
| pSOFA vs Phoenix-8 | 0.000 | −0.044 to 0.043 | 0.985 |
| pSOFA vs PELOD-2 | 0.018 | −0.041 to 0.078 | 0.544 |
| pSOFA vs PRISM III | 0.045 | −0.023 to 0.113 | 0.194 |
| PSS vs PHO-Phoenix | 0.020 | 0.002 to 0.038 | 0.034 |
| PSS vs Phoenix-8 | −0.019 | −0.044 to 0.006 | 0.140 |
| PSS vs PELOD-2 | 0.000 | −0.045 to 0.045 | 0.998 |
| PSS vs PRISM III | 0.026 | −0.036 to 0.089 | 0.407 |
| PHO-Phoenix vs Phoenix-8 | −0.038 | −0.071 to −0.006 | 0.021 |
| PHO-Phoenix vs PELOD-2 | −0.020 | −0.071 to 0.031 | 0.449 |
| PHO-Phoenix vs PRISM III | 0.007 | −0.062 to 0.075 | 0.843 |
| Phoenix-8 vs PELOD-2 | 0.019 | −0.032 to 0.070 | 0.470 |
| Phoenix-8 vs PRISM III | 0.045 | −0.014 to 0.105 | 0.136 |
| PELOD-2 vs PRISM III | 0.027 | −0.030 to 0.083 | 0.354 |

ΔAUC is the AUC of the first score minus that of the second score. Values are rounded to three decimal places; differences that round to zero are displayed as 0.000.
